# Targeting ErbB3 Receptor in Cancer with Inhibitory Antibodies from Llama

**DOI:** 10.3390/biomedicines9091106

**Published:** 2021-08-28

**Authors:** Igor E. Eliseev, Valeria M. Ukrainskaya, Anna N. Yudenko, Anna D. Mikushina, Stanislav V. Shmakov, Anastasiya I. Afremova, Viktoria M. Ekimova, Anna A. Vronskaia, Nickolay A. Knyazev, Olga V. Shamova

**Affiliations:** 1 Laboratory of Renewable Energy Sources, Alferov University, St. Petersburg 194021, Russia; annamikushina303@gmail.com (A.D.M.); stas-svs@list.ru (S.V.S.); vrons.ann@gmail.com (A.A.V.); 2 Center for Personalized Medicine, FSBSI Institute of Experimental Medicine, St. Petersburg 197376, Russia; oshamova@yandex.ru; 3 Shemyakin-Ovchinnikov Institute of Bioorganic Chemistry, Russian Academy of Sciences, Moscow 117997, Russia; ukrainskaya49@gmail.com; 4 Research Center for Molecular Mechanisms of Aging and Age-Related Diseases, Moscow Institute of Physics and Technology, Dolgoprudny 141700, Russia; yudenkoan@gmail.com; 5 CJSC Biocad, St. Petersburg 198515, Russia; afremova@biocad.ru (A.I.A.); ekimova@biocad.ru (V.M.E.); 6 Saint-Petersburg Clinical Scientific and Practical Center for Specialized Types of Medical Care (Oncological), St. Petersburg 197758, Russia; nickolayknz@gmail.com

**Keywords:** cancer therapy, monoclonal antibodies, receptor tyrosine kinases, HER3, antibody purification, conformational stability, disulfide bonds, single-domain antibodies, VHH, nanobodies

## Abstract

The human ErbB3 receptor confers resistance to the pharmacological inhibition of EGFR and HER2 receptor tyrosine kinases in cancer, which makes it an important therapeutic target. Several anti-ErbB3 monoclonal antibodies that are currently being developed are all classical immunoglobulins. We took a different approach and discovered a group of novel heavy-chain antibodies targeting the extracellular domain of ErbB3 via a phage display of an antibody library from immunized llamas. We first produced three selected single-domain antibodies, named BCD090-P1, BCD090-M2, and BCD090-M456, in *E. coli*, as SUMO fusions that yielded up to 180 mg of recombinant protein per liter of culture. Then, we studied folding, aggregation, and disulfide bond formation, and showed their ultimate stability with half-denaturation of the strongest candidate, BCD090-P1, occurring in 8 M of urea. In surface plasmon resonance experiments, two most potent antibodies, BCD090-P1 and BCD090-M2, bound the extracellular domain of ErbB3 with 1.6 nM and 15 nM affinities for the monovalent interaction, respectively. The receptor binding was demonstrated by immunofluorescent confocal microscopy on four different ErbB3^+^ cancer cell lines. We observed that BCD090-P1 and BCD090-M2 bind noncompetitively to two distinct epitopes on the receptor. Both antibodies inhibited the ErbB3-driven proliferation of MCF-7 breast adenocarcinoma cells and HER2-overexpressing SK-BR-3 cells, with the EC_50_ in the range of 0.1–25 μg/mL. BCD090-M2 directly blocks ligand binding, whereas BCD090-P1 does not compete with the ligand and presumably acts through a distinct allosteric mechanism. We anticipate that these llama antibodies can be used to engineer new biparatopic anti-ErbB3 or bispecific anti-ErbB2/3 antibodies.

## 1. Introduction

The four ErbB receptor tyrosine kinases serve as an interface to the complex signaling network with modular architecture, redundancy, and multiple feedback circuits, which regulate the essential cellular processes in the development of the epithelial cells, heart, nervous system, and mammary gland [1]. Aberrant signaling by EGFR (ErbB1) and ErbB2, caused by gene amplification, deletions in the extracellular domain, activating kinase domain mutations, or ligand coexpression, drives the progression of many human cancers, especially in the breast, lungs, and colon [2]. Signal transduction by ErbBs starts from ligand binding, which induces extracellular domain rearrangement, leading to receptor dimer stabilization and allosteric activation of the intracellular tyrosine kinase domain. However, the following two of the four receptors are not autonomous: ErbB2 does not interact with ligands [3] and ErbB3 lacks tyrosine kinase activity [4], which restricts their operation to heterodimers with other ErbBs. Particularly, the response of ErbB3 to its ligand, heregulin, proceeds through ErbB2-mediated transactivation occurring in ErbB2/ErbB3 heterodimers [5]. The proliferation of ErbB2-overexpressing breast cancer cells requires ErbB3, indicating that the ErbB2/ErbB3 heterodimer acts as a potent oncogenic unit [6], which couples to the downstream Ras/MAPK pathway through a single ErbB2 C-terminal phosphorylation site [7], and to the PI3K/Akt pathway through six ErbB3 C-terminal phosphorylation sites [8]. Strikingly, ErbB3 not only participates in oncogenic signaling, but also confers resistance to ErbB2 tyrosine kinase inhibitors via a negative feedback circuit, which maintains the activity of the PI3K/Akt pathway [9,10]. The pharmacological inhibition of ErbB3 by a monoclonal antibody sensitizes ErbB2-overexpressing cancer cells and xenografts to a tyrosine kinase inhibitor, showing that ErbB3 is an important cancer therapeutic [10].

The mechanisms of ErbB3-mediated resistance to anti-ErbB1/2 monoclonal antibodies, tyrosine kinase inhibitors, and hormonal therapy, and different approaches to its therapeutic targeting in cancer, have been extensively reviewed [11,12,13].

The principal strategy is to target the ErbB3 extracellular domain with a monoclonal antibody, which can lock it in a tethered auto-inhibited conformation, block ligand binding, prevent dimerization with other ErbBs, induce receptor internalization, or engage immune effector cells, leading to antibody-dependent cellular cytotoxicity. Several antibodies that are currently in clinical studies attack different epitopes on the receptor, and therefore possess unique pharmacological characteristics and mechanisms of action. Elgemtumab (LJM716) binds domains 2 and 4, and locks ErbB3 in inactive conformation, thus inhibiting both ligand-dependent and ligand-independent proliferation of various cancer cell lines, without direct interference with heregulin binding [14]. Lumretuzumab (RG7116) binds domain 1, blocks ligand binding, and prevents ErbB3 phosphorylation, while also downregulates the membrane surface receptor and induces antibody-dependent cellular cytotoxicity in mouse xenografts [15]. Another antibody KTN3379 inhibits both ligand-dependent and ligand-independent ErbB3 signaling by a unique allosteric mechanism; it binds domain 3 and the 2/3 hinge region, which blocks the conformational rearrangement that is necessary for ligand binding and receptor dimerization, and arrests the extracellular domain in a tethered state [16]. 

We took a different approach and used heavy-chain antibodies from llama to target the extracellular domain of ErbB3. Discovered in the serum of camel [17], heavy-chain antibodies have found many applications in research and medicine. Unlike classic immunoglobulins, these molecules are naturally devoid of light chains, and their variable fragment, also called VHH or nanobody, is a single immunoglobulin domain of 12–15 kDa size. These single-domain antibody fragments possess many advantages, including high solubility, stability, and facile biotechnological production [18]. The genetic and structural features of llama antibodies generate an unusual antigenic repertoire with a broad range of epitopes, differing from conventional antibodies [19,20]. Numerous therapeutic applications of single-domain antibodies have been reviewed recently [21]. Besides medicine, other uses as research tools in cellular biology [22], or crystallization chaperones in structural biology [23], are becoming common. Here, we report the discovery, biochemical characterization, and biological activity of three new anti-ErbB3 llama single-domain antibodies, named BCD090-P1, BCD090-M2, and BCD090-M456, which were discovered by a phage display of an antibody library from llamas immunized with the extracellular fragment of human ErbB3 [24]. 

## 2. Materials and Methods

### 2.1. Materials

All chemicals for the preparation of buffers, IPTG, and *L*-rhamnose were purchased from Panreac (Applichem); SDS-PAGE and Western blot reagents were from Bio-Rad and HisProbe-HRP conjugate was from Thermo Scientific. PCR reagents, restriction enzymes, and PNGase F were from New England Biolabs. The TB and 2xYT media were prepared from BD Bacto tryptone and BD Bacto yeast extract. HyCell TransFx-C media, HyClone DMEM and HyClone fetal bovine serum were purchased from GE Healthcare, recombinant human heregulin-1β was purchased from BioVision (cat. 4711), trastuzumab and rituximab (reditux) were from Roche and Dr. Reddy’s. Membrane filters and Amicon centrifuge concentrators were purchased from Millipore. For IMAC we used cOmplete chromatography columns (Roche), HisTrap HP columns (GE Healthcare), and Ni-NTA resin (Qiagen). Affinity CaptureSelect C-tag resin was from Thermo Fisher. All other chromatography columns, MonoQ 10/100 and MonoS 5/50, Superdex 75 Increase 10/300, HiLoad 16/600 Superdex 200pg, and LMW calibration standards were purchased from GE Healthcare. For cleavage, we used TEV protease mutant TEVpM2 [25], which we expressed and purified as described in Supplementary Information. Ellman reagent (cat. D8130) and Hoechst 33342 were purchased from Sigma, AF488 and sulfo-cyanine5 NHS esters were from Lumiprobe and MTS reagent was from BioVision (cat. 2808). All coupling reagents and sensor chips for surface plasmon resonance were purchased from GE Healthcare.

### 2.2. Expression Vectors

All expression vectors used in this study are summarized in Table S1, their maps and protein sequences are given in Figure S1 and Table S2. For the cytoplasmic bacterial expression of the single-domain antibodies, we used pSolSUMO vector (Lucigen). The PCR-amplified fragments encoding the antibodies were mixed with the linearized pSol vector and used to transform *E. cloni* 10G cells (Lucigen). For the periplasmic expression, we used pET22b vector (Novagen) bearing N-terminal pelB leader peptide and C-terminal histidine tag. The gene fragment encoding BCD090-P1 was amplified with specific primers overlapping the vector and incorporated by overlap-extension PCR. To produce ErbB3 receptor fragments ECD (residues 21–643) and ECD^III^ (residues 329–532), we used pEE vector with hCMV promoter and Igκ leader designed for transient secretional expression in CHO cells. The gene fragment encoding ECD was cloned into the pEE vector using SalI and NotI restriction sites. The gene encoding ECD^III^ was amplified and fused with C-terminal His_6_ and EPEA tags and cloned into the pEE vector SalI and BamHI restriction sites. The plasmids were produced in *E. coli* XL1-Blue cells (Stratagene), purified and sequenced.

### 2.3. Cytoplasmic Expression of Single-Domain Antibodies

Chemically competent *E. coli* SHuffle T7 express cells (NEB) or *E. coli* BL21 (DE3) cells (NEB) were transformed by each pSol vector, and single colonies were used to start small-scale overnight cultures. The next day 1-liter bacterial cultures were inoculated by 1:100 volume of overnight culture and grown in TB (2.0% tryptone, 2.4% yeast extract, 0.4% glycerol, 17 mM KH_2_PO_4_, 72 mM K_2_HPO_4_) with 50 μg/mL kanamycin at 30 °C. At OD 0.9–1.1 protein expression was induced by the addition of *L*-rhamnose to a final concentration 10 mM, temperature was lowered to 25 °C and cells were grown overnight for additional 18–20 h. Cells were harvested by 5 min centrifugation at 10,000× *g* and resuspended in IMAC buffer (50 mM Na_2_HPO_4_ pH 8.0, 0.3 M NaCl) with 1 mM PMSF, 1 mM EDTA, and 1 mM benzamidine as protease inhibitors. Periplasmic expression of BCD090-P1 is described in Supplementary Information.

### 2.4. Purification and TEV Cleavage of the Single-Domain Antibodies

Cells were lysed by ultrasonication on ice, and cell debris were pelleted by 20 min centrifugation at 40,000× *g*. Then proteins were precipitated by slowly adding ammonium sulfate to 70% saturation with continuous mixing at 4 °C. The precipitate was pelleted and dissolved in IMAC buffer, filtered through a 0.22 μM membrane and loaded on a pre-equilibrated IMAC column. The target protein was eluted by IMAC buffer with 0.3 M imidazole and analyzed by SDS-PAGE. To cleave the fusion protein, samples were dialyzed against TEV buffer (30 mM Tris pH 8.0, 0.5 mM EDTA, 1 mM DTT), mixed with TEVpM2 protease at 1:50 enzyme-to-substrate ratio and incubated for 4 h at room temperature with mild agitation. Histidine-tagged SUMO and TEV were then removed by negative IMAC chromatography in batch mode using Ni-NTA agarose beads. Polishing ion-exchange chromatography was performed on an NGC Discover system (Bio-Rad). Samples were dialyzed against 20 mM Tris pH 8.0 (MonoQ), or 20 mM sodium acetate pH 6.0 (MonoS). The target protein was eluted by a 0–0.6 M NaCl gradient. Fractions of ½ column volume each were collected, analyzed by SDS-PAGE, then pooled, dialyzed overnight against 20 mM HEPES pH 7.5, 50 mM NaCl and concentrated using centrifuge filters. All protein concentrations were measured spectrophotometrically with the parameters given in Table S3, calculated with the ProtParam tool [26]. 

### 2.5. Expression and Purification of ErbB3 ECD and ECD^III^

The two fragments of the ErbB3 receptor were produced in CHO-T-HC cells transiently transfected by polyethyleneimine. For each protein, one liter of cell culture was grown in flasks in HyCell TransFx-C media at 32 °C for eight days. Culture fluid was clarified by sterile filtration through Opticap XL capsule filters and used for protein purification. The ECD was purified by IMAC on a HisTrap column followed by preparative size exclusion chromatography on a Superdex 200 pg column. For IMAC, clarified culture fluid was supplemented with 1 mM NiCl_2_ and 10 mM imidazole, and loaded on a column. The protein was eluted by IMAC buffer with 0.3 M imidazole. The eluate was loaded on a Superdex 200 pg column pre-equilibrated with PBS, and the peak fractions from size exclusion chromatography were pooled and concentrated on a centrifuge filter. The ECD^III^ fragment was purified from culture fluid by affinity chromatography on a CaptureSelect C-tag resin, which utilized EPEA tag. The protein was eluted by 3 mL of 20 mM sodium citrate pH 3.0 and immediately neutralized by adding 0.3 mL 1 M Tris pH 8.0, 1 M NaCl. The purified ECD^III^ was dialyzed against PBS and filtered through a 0.22 µm membrane.

### 2.6. Gel Electrophoresis and Western Blotting

Soluble cell extracts were diluted to normalize the total protein concentration measured by Bradford assay. Purified ErbB3 receptor fragments were deglycosylated with PNGase F under denaturing conditions prior to analysis. SDS-PAGE was performed under reducing conditions using Mini-Protean system (Bio-Rad) according to the standard protocol. For Western blotting, proteins were transferred onto a nitrocellulose membrane with Trans-Blot Turbo system (Bio-Rad) and developed with HisProbe-HRP conjugate directed against histidine tags. Chemiluminescence was detected with ChemiDoc imaging system (Bio-Rad).

### 2.7. Size Exclusion Chromatography

Chromatography was performed on an Acquity UPLC system (Waters) using a Superdex 75 column. The column was equilibrated with PBS pH 7.4 and calibrated by a mixture of LMW protein standards. The samples were diluted to 0.5–0.9 mg/mL, and 100 µL of each sample were injected to a pre-equilibrated column. The chromatograms were recorded as UV absorbance at 280 nm. Calibration with LMW standards was used to estimate molecular weights for antibody peaks from their elution volumes. 

### 2.8. Circular Dichroism Spectroscopy

Circular dichroism spectra were measured with a Chirascan spectrometer (Applied Photophysics) in the range 190–250 nm with 1.0 nm step size. Samples were prepared by the dilution of antibody stock solutions to 0.3–0.4 mg/mL in a low UV absorbance buffer (5 mM Na_2_HPO_4_–KH_2_PO_4_ pH 7.5, 100 mM NaF). Each spectrum was averaged over ten measurements, converted to mean residue Δε units (M^−1^cm^−1^), and then deconvoluted using the β-structure selection method [27] recently implemented in BeStSel web server [28]. 

### 2.9. Urea Denaturation Curves

Urea denaturation curves were measured according to a standard protocol [29] with intrinsic tryptophan fluorescence used as a reporter signal. Series of protein samples were prepared in 50 mM Na_2_HPO_4_ with urea concentration ranging from zero to 10 M in 0.25 M increments. After overnight incubation, fluorescence spectra were acquired at 20 °C with a Chirascan spectrometer (Applied Photophysics) in 300–500 nm range with 280 nm excitation. The collected series of intrinsic fluorescence spectra were used to calculate the skewness of *I/λ* distribution, which was used as a characteristic of a red-shift of emitted light. The values were plotted versus urea concentration, normalized, and fitted with a sigmoid curve describing the standard two-state protein denaturation model.

### 2.10. Ellman Assay

The quantitation of free sulfhydryl groups was performed by Ellman reagent using molar extinction coefficient for the leaving group (NTB) of 13700 M^−1^cm^−1^ at 412 nm. Protein was diluted in the denaturation buffer (50 mM Na_2_HPO_4_–NaH_2_PO_4_ pH 7.4, 8 M urea, 1% SDS, 1 mM EDTA) to a final concentration of 50–100 μM, then an excess of Ellman reagent was added, and absorbance at 412 nm was read over 30 min period. The maximum signal was reference-subtracted, divided by protein concentration, and averaged over three replicates. To measure the number of disulfide bonds experimentally, we used an assay with 2-nitro-5-thiosulfobenzoate (NTSB) [30]. The denatured protein was reduced by an excess of sodium sulfite, and total number of cysteines was measured by reaction with NTSB, which has the same leaving group as Ellman reagent. The NTSB was synthesized from Ellman reagent by reaction with sodium sulfite and O_2_, as described in the original paper [30]. The protein solution was mixed with freshly prepared denaturing buffer (0.1 M glycine–NaOH pH 9.4, 6 M guanidine thiocyanate, 0.2 M Na_2_SO_3_, 5 mM EDTA) and NTSB, afterwards the analysis was performed as described for Ellman assay.

### 2.11. Mass Spectrometry

Antibody stock solutions were diluted to 1–2 mg/mL and thoroughly dialyzed against mQ water. Acetic acid (BCD090-P1) or formic acid (all other samples) were added to protein solutions to a final concentration of 1%. Mass spectra were measured on a maXis Q-TOF spectrometer (Bruker Daltonik) in positive ion mode using direct injection with electrospray ionization. Mass spectra were averaged using DataAnalysis 4.0 software (Bruker Daltonik), and then exported as text files containing m/z versus intensity. All further analysis, namely, deconvolution of charge states and fitting of the isotopic distributions, was performed according to the methodology developed by Rhoads et al. [31] in Matlab using their script, which is available in Supplementary Materials for the paper [31]. 

### 2.12. Surface Plasmon Resonance

All surface plasmon resonance experiments were performed on a Biacore T200 instrument (GE Healthcare) using CM5 sensor chips at 37 °C. Either ErbB3 ECD or ECD^III^ were immobilized on a chip surface via amine coupling. Antibody solutions were serially diluted in HBS (10 mM HEPES pH 7.4, 150 mM NaCl) with 50 µg/mL BSA to yield concentration series. Acquired sensograms were reference-subtracted and analyzed in Biacore Evaluation software (GE Healthcare). Equilibrium binding response was read at 5 s before injection end and fitted by the Hill equation. Kinetic rate constants k_a_ and k_d_ were obtained by fitting the sensograms globally by simple 1:1 binding model in Biacore Evaluation at three different concentrations near K_D_. The reported parameters are geometric means from two independent experiments. 

Epitope mapping and ligand competition utilized in tandem experimental design [32]. The first antibody or ligand was injected into a chip for 10 min to saturate receptors, followed by the 10 min injection of a mixture of both antibodies or antibody plus ligand. The concentrations of antibodies were high compared to the corresponding K_D_ – 0.5 µM for BCD090-P1 and M2, 16 µM for BCD090-M456, heregulin concentration was 0.1 μM. The experiments were repeated with a different order of the analytes.

### 2.13. Cell Proliferation Assays

The human breast adenocarcinoma MCF-7 and BT-474, hepatocellular carcinoma Hep G2, K562 and HeLa cell lines were obtained from the bank of cell cultures of the Institute of Cytology of the Russian Academy of Sciences, the SK-BR-3 breast adenocarcinoma cell line was provided by CJSC Biocad. Cells were cultured in DMEM supplemented with 10% fetal bovine serum and 40 μg/mL gentamicin at 37 °C in a humidified atmosphere with 5% CO_2_. Two weeks before the experiment MCF-7 cells were transferred to DMEM without phenol red with charcoal-stripped fetal bovine serum (CSS) prepared by treatment with 5% dextran-coated activated charcoal as previously described [33]. 

For the assay, cells were grown until 50–70% confluence, collected and resuspended in DMEM with 0.3% CSS (MCF-7) or full serum (SK-BR-3) and 50 ng/mL recombinant human heregulin-1β. Cell suspension with at least 90% viability was diluted and placed in a 96-well flat-bottom plate, giving approximately 5000 cells per well. The next day sterile filtered antibody samples were serially diluted in assay media and added to the wells, assay media without an antibody, rituximab, and HeLa cells expressing negligible amounts of ErbB2 and ErbB3 receptors were used as negative controls. Culture plates were incubated for 5 days at 37 °C in a humidified atmosphere with 5% CO_2_, then cell proliferation was measured by adding MTS reagent. After incubation at 37 °C, absorbance at 495 nm was read by Multiskan GO plate spectrophotometer (Thermo Scientific). Half maximal effective concentration EC_50_ was then obtained by fitting the obtained dose–response curve by the Hill equation. The growth curves were measured in the same way. To assess cell viability by flow cytometry, 25,000 cells were seeded in a 24-well plate and incubated as described above. On the fourth and seventh days, cells were harvested by washing with 0.25% trypsin, 2 mM EDTA and stained with propidium iodide. Then cell suspension was analyzed on a BD FACSCanto cytometer. The reported data represent the mean of three replicates ± SD. Cell viability changes were analyzed by *t*-test.

### 2.14. Immunocytochemistry and Confocal Microscopy

Single-domain antibody and trastuzumab were directly labeled by sulfo-Cy5 or AF488 according to standard protocol [34]. Briefly, protein solutions in 0.1 M Na_2_HPO_4_ pH 8.2 were mixed with excess of fluorescent dye NHS ester and incubated for 2 h at +4 °C. The unreacted dye was removed by buffer exchange on a Sephadex G25 column pre-equilibrated with PBS. The labeling efficiency was analyzed by measuring absorbance at 280 nm and 646 nm (Cy5) or 495 nm (AF488). Two days prior to experiment, 2 × 10^5^ cells were seeded onto Petri dishes with cover slips. K562 cells were transferred onto coverslips after staining. After incubation under growing conditions, cells were washed twice with PBS, stained for 30 min in PBS with 5 μg/mL labeled antibodies and Hoechst 33342, and rinsed with PBS. Images were obtained on AxioObserver Z1 confocal microscope (Carl Zeiss).

## 3. Results

### 3.1. Expression and Purification of Single-Domain Antibodies

Recombinant single-domain antibodies are produced in all kinds of expression hosts [35]. Traditionally, single-chain antibodies were expressed in bacterial periplasm [36], because of its natural oxidizing environment, conducive to disulfide bond formation. This strategy is still frequently used for both single-domain antibodies [37] and single-chain antibodies derived from classical Fabs [38]. However, it seems more appealing to produce them in the oxidative cytoplasm of engineered *E. coli* strains, because of its larger volume compared to periplasm and the absence of a limiting translocation stage.

**Figure 1:**
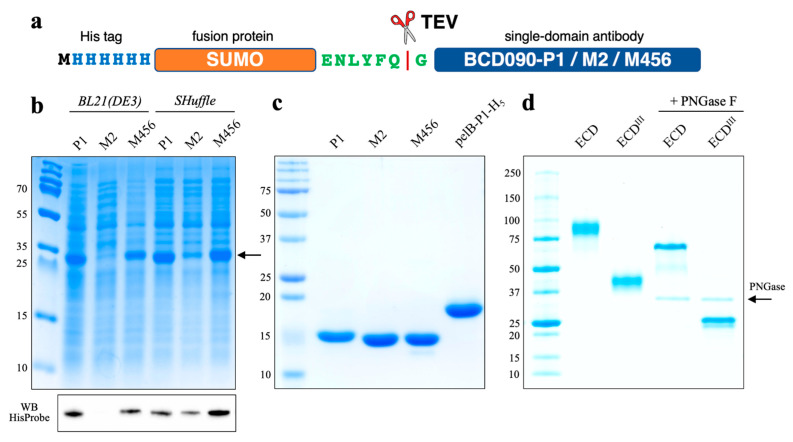
Expression and purification of the anti-ErbB3 single-domain antibodies and ErbB3 receptor fragments. (**a**) Scheme of SUMO fusions used to produce single-domain antibodies; (**b**) SDS-PAGE and Western blot of soluble extracts, the recombinant fusion proteins have MW 27 kDa; BCD090-M2 expressed in soluble form only in SHuffle cells, but not BL21(DE3); (**c**) SDS-PAGE of the purified single-domain antibodies, periplasmically expressed pelB-BCD090-P1-His_5_ migrates higher due to the presence of uncleaved pelB leader and histidine tag; (**d**) SDS-PAGE of the purified human ErbB3 extracellular domain (ECD, MW 75 kDa) and the extracellular subdomain 3 (ECD^III^, MW ~25 kDa); receptor fragments analyzed in the native glycosylated form and after treatment with PNGase F.

For the cytoplasmic expression of single-domain antibodies, we used N-terminal histidine-tagged SUMO fusions, connected to a target protein through a TEV recognition sequence. We used SUMO because of its small size (11 kDa), robust folding, and the previous report of its advantages [39]. Antibodies were expressed in SHuffle T7 express cells, a *trxB gor* suppressor *E. coli* strain that allows the formation of disulfide bonds in the cytoplasm [40], or a standard expression strain BL21(DE3). All three fusion proteins were efficiently expressed in soluble form in SHuffle cells, as observed in Figure 1. The production of BCD090-M2 appeared to be the most difficult and failed in BL21(DE3), where the protein was located entirely in the inclusion bodies. The yields of the fusion proteins after purification (see Figure S2 and Table S4) ranged from 50 to 180 mg per liter of culture, which is comparable to the highest reported in the literature, e. g., by Zarschler et al. [41], where 120–175 mg of recombinant anti-EGFR single-domain antibodies were purified from one liter of *E. coli* SHuffle culture. On the contrary, the classic method of periplasmic production with a pET22 vector usually yielded only ∼10 mg of pure antibody per liter of culture and was complicated by inefficient cleavage of the leader peptide.

### 3.2. Characterization by Analytical Size Exclusion Chromatography

**Figure 2:**
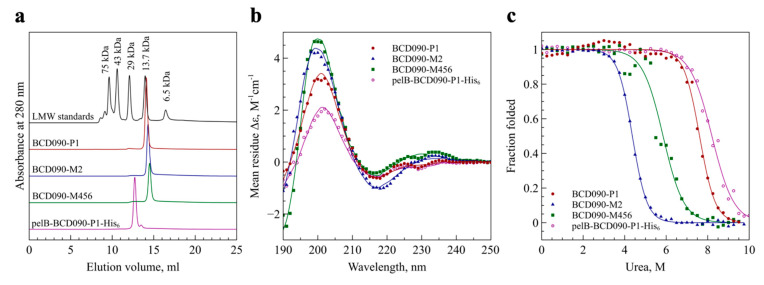
Biochemical characterization of the anti-ErbB3 single-domain antibodies. (**a**) Size exclusion chromatography shows that all antibodies are present as monomers of expected molecular weight; pelB-BCD090-P1-His_5_ elutes earlier due to the presence of the leader peptide and a histidine tag; (**b**) circular dichroism spectroscopy of the single-domain antibodies, experimental data points are shown as symbols and fitted curves shown as lines. Deconvolution of the spectra gives a secondary structure with prevalent antiparallel β-sheets and turns, typical of immunoglobulin fold; (**c**) experimental urea denaturation curves of the single-domain antibodies (symbols) and their fits with the two-state folding model (lines). All three antibodies show remarkable stability with denaturation midpoints above 4 M urea.

We analyzed the purified single-domain antibodies by high-resolution size exclusion chromatography under native conditions to determine their aggregation state. As observed in Figure 2a, all three proteins elute as single symmetrical peaks near the 13.7 kDa standard, ribonuclease A. This evidence that the cytoplasmically expressed and processed antibodies present in a folded, monomeric form. However, BCD090-P1 purified from the periplasm has an estimated molecular weight of ~22 kDa, as calculated from a calibration curve. This we attribute to the presence of the uncleaved pelB leader and a histidine tag. Being unstructured, these peptides increase the antibody hydrodynamic radius compared to globular proteins of the same molecular weight, which results in earlier elution from the column.

### 3.3. Secondary Structure Analysis by Circular Dichroism Spectroscopy

To confirm the correct folding of the recombinant single-domain antibodies, we studied their secondary structure by circular dichroism spectroscopy. The analysis of the circular dichroism spectra of β-structured proteins is challenging, due to the great spectral variability arising from the structural diversity of the different forms of β-sheet structures. We employed a new approach called the β-structure selection method [27], designed specifically to analyze β-structured proteins. As seen from Figure 2b, the deconvolution of the spectra produced a perfect fit to the experimental data with 2–3% normalized root mean square deviation. For all the antibodies, the analysis revealed that the structure was composed principally of antiparallel β-sheets and β-turns with a minor proportion of parallel β-sheets and zero helical content. The estimated β-sheet/β-turn content was 57/10 for BCD090-P1, 55/8 for BCD090-M2, 53/9 for BCD090-M456, and 54/11 for pelB-BCD090-P1-His_5_, which closely resemble the values that are typically obtained for immunoglobulin fold proteins, e.g., standard Fabs [42], and consistent with our previous crystal structures of BCD090-M2 [43]. The fold recognition by BeStSel unequivocally assigns the correct β-sandwich architecture and immunoglobulin/jelly roll topology to all the samples.

### 3.4. Analysis of Conformational Stability

One of the decisive advantages of single-domain antibodies is their superb conformational stability. We therefore analyzed their folding thermodynamics by studying urea denaturation curves that were obtained by spectroscopy of tryptophan fluorescence. As we experienced earlier [44], the intrinsic fluorescence of single-domain antibodies is sensitive to the microenvironment inside the protein’s hydrophobic core, and so appears a perfect reporter in folding and denaturation studies. The data for all the antibodies, shown in Figure 2c, conformed to the standard two-state model and demonstrated their excellent resistance to denaturation. The fitting of the experimental data allowed us to estimate unfolding free energy changes in the absence of denaturant (ΔG), which are given in Table S5. BCD090-P1, both untagged and bearing a leader and a histidine tag, showed a striking stability with half denaturation occurring near 8 M urea and ΔG of 11.7 kcal⋅mol^−1^.

### 3.5. Free Thiol and Disulfide Quantitation by Ellman Reagent and NTSB

Having established that our single-domain antibodies are monomeric and possess a β-sheet structure, we examined them biochemically to determine the oxidation state of the two cysteines Cys_22_ and Cys_92_. In IgGs, these highly conserved cysteines form a buried intramolecular disulfide bond which connects B and F strands at the beginning of the CDR H1 and CDR H3 loops. We used two complementary experiments with the Ellman reagent and NTSB to quantitate the free sulfhydryl group (S–H) and disulfide bonds (S–S), respectively. The results of the experiments are given in Table S6. Both the antibodies BCD090-P1 and BCD090-M2 have a single disulfide bond and no detectable free sulfhydryl groups, as expected for the native conformation. Surprisingly, BCD090-M456 does not have a disulfide bond, because a single S–H group was detected by the Ellman reagent. The presence of only one free sulfhydryl per molecule is inconsistent with the size exclusion chromatography, where we clearly observed that the antibody is monomeric. One interpretation of the experimental data is that both cysteines in BCD090-M456 are indeed reduced, but one of them is not detectable in the Ellman assay, due to steric or electrostatic constraints, as often observed [45,46]. Unexpectedly, the absence of the conservative disulfide bond does not prevent this antibody from having the correct immunoglobulin fold and remarkable conformational stability.

### 3.6. Mass Spectrometry Analysis of the Intact Mass and Oxidation State

**Figure 3:**
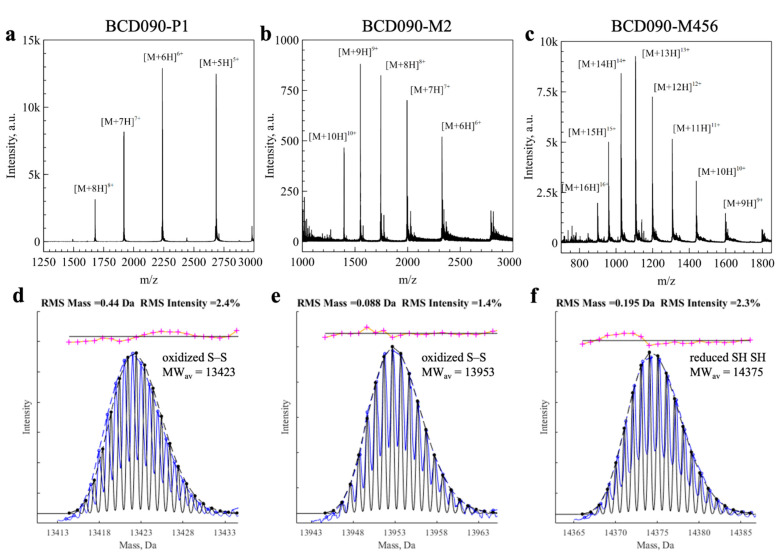
Analysis of the single-domain antibodies by ESI-MS. (**a**–**c**) Experimental mass spectra with individual charge states indicated; (**d**–**f**) experimental isotopic distribution after charge deconvolution (blue) fitted with theoretical isotopic distribution (black). For BCD090-P1 and BCD090-M2, best fit obtained with the formula corresponding to oxidized cysteines (-2H) as expected. On the contrary, experimental data for BCD090-M456 were best fitted with formula with free cysteines.

We then measured the molecular masses of the intact single-domain antibodies and studied their molecular heterogeneity by mass spectrometry (Figure 3). After deconvolution of the charge states, we aimed to analyze small mass differences due to the formation of disulfide bonds (2 Da) and other post-translational modifications, i.e., deamidation (1 Da). Since the direct analysis of intact mass is hindered by natural isotopic distribution, the general strategy is to use fragmentation methods and then study the MS2 spectra of the generated peptides [47]. We took a different approach, developed by Rhoads et al. to distinguish copper and zinc bound to superoxide dismutase [31]. In this method, theoretical isotopic distribution is calculated for each empirical formula under consideration and then used to fit the measured isotopic distribution.

After testing this approach for the disulfide bonds in lysozyme (see Figure S3), we applied it to single-domain antibodies. In Figure 3d–f we show the experimental isotopic distributions (blue) fitted with theoretical curves (black). For BCD090-P1 and BCD090-M2, the best fits were obtained with empirical formulas with a disulfide bond (-2H). For BCD090-M456, the experimental data agreed with the formula lacking a disulfide bond, which is consistent with biochemical experiments. Besides the disulfide bond analysis, mass spectrometry also confirms complete and accurate cleavage by TEV protease and the absence of other post-translational modifications. Remarkably, the analysis of the oxidation state was run on a regular time-of-flight spectrometer and did not require any chemical derivatization of the thiol groups.

### 3.7. Affinity and Kinetics of Binding to ErbB3 ECD and ECD^III^

**Figure 4:**
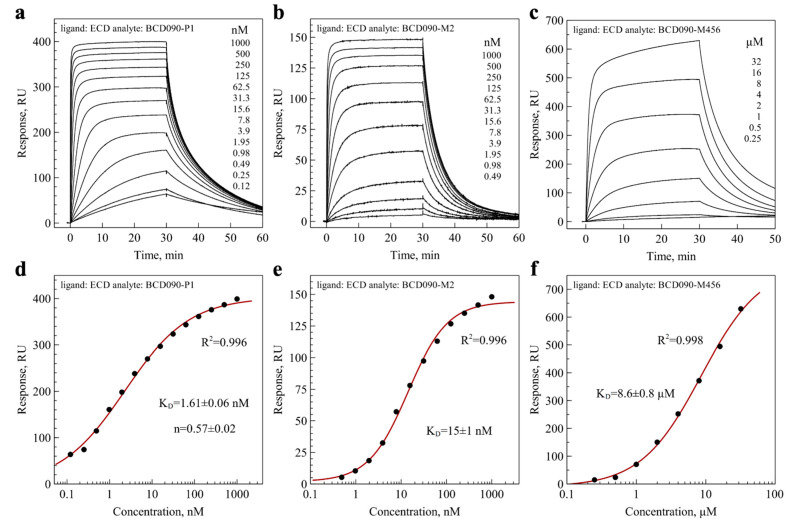
Binding of the single-domain antibodies to the ErbB3 extracellular domain analyzed by surface plasmon resonance. (**a**–**c**) Experimental binding sensograms after reference subtraction; (**d**–**f**) equilibrium binding response plotted versus concentration on semi-logarithmic scale, experimental points shown as dots and fits with the Hill equation shown as lines. Data for BCD090-M2 were previously reported in our paper [43].

After the characterization of the single-domain antibodies, we studied their binding to immobilized ErbB3 ECD by surface plasmon resonance. The representative series of experimental sensograms are presented in Figure 4a–c, and the data for pelB-BCD090-P1-His_5_ are shown in Figure S4. The antibodies did not exhibit any cross-reactivity with the extracellular domain of ErbB2, as observed in Figure S5. For the equilibrium analysis, we plotted the binding response versus the antibody concentration, and fitted the data with the Hill equation to obtain K_D_ (panels d–f). Both BCD090-P1 and BCD090-M2 had equilibrium dissociation constants in the nanomolar range, while BCD090-M456 had a lower affinity. All the experimental data conformed very well with the model with R^2^ > 0.99. The same experimental data were used for kinetic analysis, which yielded the kinetic rate constants k_a_ and k_d_, and a complementary measurement for the equilibrium constant K_D_. All these values are summarized in Table 1. As observed from the table, the kinetic and equilibrium analyses give remarkably close values for affinities of the antibodies towards ErbB3 ECD. BCD090-P1 is the most potent binder, and its affinity is slightly (four-fold) decreased in the presence of the N-terminal pelB leader peptide. Surprisingly, while BCD090-M456 has the lowest affinity among the three antibodies, it forms the most stable complex with the receptor with the lowest dissociation constant. Three orders of magnitude difference in the equilibrium constant with other antibodies arises entirely from its very low association rate constant. The slow association can be either due to the low abundance of a particular conformation that is necessary for binding (conformational selection), or due to slow isomerization (induced fit), but the data do not allow to distinguish these mechanisms. We anticipate that the molecular cause of such an unusual behavior may be the lack of a conserved disulfide bond Cys_22_–Cys_92_ or the considerable length of CDR H3 (23 residues) in this antibody.

### 3.8. Epitope Mapping and Competition with Heregulin

**Figure 5:**
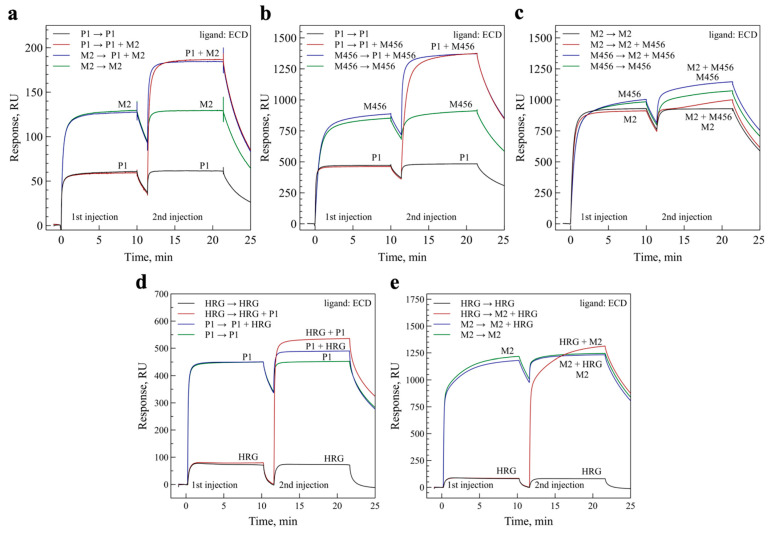
Epitope mapping by pairwise binding experiments and ligand competition. (**a**) Antibodies BCD090-P1 and BCD090-M2 bind simultaneously to ErbB3 ECD; (**b**) the same result is for BCD090-P1 versus BCD090-M456; (**c**) BCD090-M2 and BCD090-M456 attack the overlapping epitopes as seen from the lack of simultaneous binding; (**d**) BCD090-P1 does not block the binding of natural ErbB3 ligand, heregulin; (**e**) saturation of ECD with BCD090-M2 completely abolishes ligand binding.

We then performed epitope mapping experiments to elucidate the epitopes of the antibodies on the ErbB3 receptor. We first conducted SPR experiments with the ECD^III^ fragment of the extracellular domain, and observed that BCD090-P1 bound it with an almost identical affinity as the intact ECD (see Figure S6). Two other antibodies did not interact with ECD^III^ to any measurable extent. Next, we conducted pairwise binding experiments with ECD, which are shown in Figure 5. As observed from the panels (a) and (b), the binding of BCD090-P1 proceeds mutually independently with BCD090-M2 or BCD090-M456. On the contrary, BCD090-M2 and BCD090-M456 compete for the receptor, from which we conclude that they have overlapping epitopes. Similarly designed experiments were performed to examine whether the antibodies block ligand binding. Heregulin alone bound the immobilized ECD with nanomolar affinity, yet a lower equilibrium response compared to the single-domain antibodies, due to its small molecular weight. The injection of heregulin mixed with BCD090-P1 resulted in a clear increase in the equilibrium response, relative to the antibody or heregulin alone, which indicates that BCD090-P1 does not block ligand binding (see Figure 5d). In contrast, the saturation of receptors with BCD090-M2 completely abolished heregulin binding (Figure 5e). When receptors were pre-treated with heregulin, BCD090-M2 associated slower, which we attribute to the gradual displacement of the bound ligand. The strong competition of BCD090-M2 with the ligand, and the lack of binding to ECD^III^, let us conclude that this antibody and BCD090-M456 presumably bind to the subdomain 1 of ECD.

### 3.9. Antiproliferative Action on ErbB3^+^ Cancer Cells

**Figure 6:**
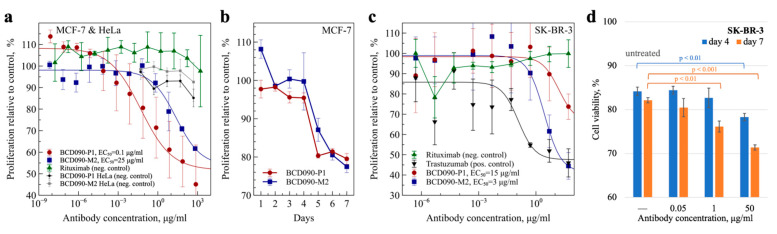
Inhibition of ErbB3-driven proliferation of cancer cells. (**a**) The antiproliferative action of BCD090-P1 or BCD090-M2 on MCF-7 cells, rituximab and HeLa cells expressing negligible amounts of ErbB3 were used as negative controls; (**b**) the growth curves of MCF-7 cells treated with 50 μg/mL single-domain antibodies; (**c**) antiproliferative action of BCD090-P1, BCD090-M2 or trastuzumab on SK-BR-3 cells; (**d**) the viability of SK-BR-3 cells treated with a mixture of the two single-domain antibodies. The dose–response curves were fitted by the Hill equation. All data represent the mean of three replicates ± SD, *p*-values were calculated using *t*-test.

The principal functional effect arising from the targeted inhibition of ErbB3 signaling is reduced growth and survival of cancer cells. The antiproliferative action of the antibodies was analyzed in vitro, using two classical models of ErbB3-overexpressing breast adenocarcinomas: MCF-7 cells and SK-BR-3 cells, which also overexpress ErbB2. Since MCF-7 cells are hormone responsive, we used charcoal-stripped serum and media without phenol red, which is a weak estrogen receptor agonist [48], to avoid assay interference from the stimulation of estrogen receptors. As seen from Figure 6a, both BCD090-P1 and BCD090-M2 inhibited the heregulin-driven proliferation of MCF-7 cells, with an EC_50_ of 0.1 and 25 μg/mL, respectively. The higher potency of BCD090-P1 compared to BCD090-M2 may arise from the higher receptor affinity and potentially from the distinct mechanism of action. While BCD090-M2 competes with heregulin directly, BCD090-P1 targets subdomain 3, which is essential for conformational rearrangement after ligand binding and therefore presumably acts through an allosteric mechanism like KTN3379 [16]. The growth curves (Figure 6b) measured at an effective concentration (50 μg/mL) show a steady decline in cancer cell viability with time, which progresses identically for the two antibodies. A similar inhibitory effect was observed in a different model with SK-BR-3 cells (Figure 6c). Indeed, the activities of anti-ErbB3 single-domain antibodies (EC_50_ 15 μg/mL and 3 μg/mL) were weaker compared to the anti-ErbB2 therapeutic antibody trastuzumab (EC_50_ 0.1 μg/mL), because of the exceptional overexpression of ErbB2 in these cells and its principal role in the stimulation of their growth. When SK-BR-3 were treated with a mixture of the two antibodies BCD090-P1 and BCD090-M2, a decrease in cancer cell viability was observed at 1 μg/mL (Figure 6d). Interestingly, in this model BCD090-M2 exhibited higher activity than BCD090-P1, which evidence that the two mechanisms of inhibition have variable effects on cancer cells with different phenotypes. The noncompetitive binding of the two antibodies and their ability to inhibit proliferation via distinct mechanisms suggests that their simultaneous action may be synergistic. It was previously demonstrated that the binding of two noncompetitive antibodies to extracellular domains of EGFR [49] and ErbB2 [50] enhances inhibition, primarily by stimulating receptor endocytosis. The same strategy was applied to ErbB3 but resulted in a relatively small improvement in the inhibitory effect [51]. It remains obscure whether these differences originate from the different mechanisms of receptor endocytosis or from the characteristics of particular antibodies.

### 3.10. Immunofluorescent Staining of ErbB3^+^ Cancer Cells

**Figure 7:**
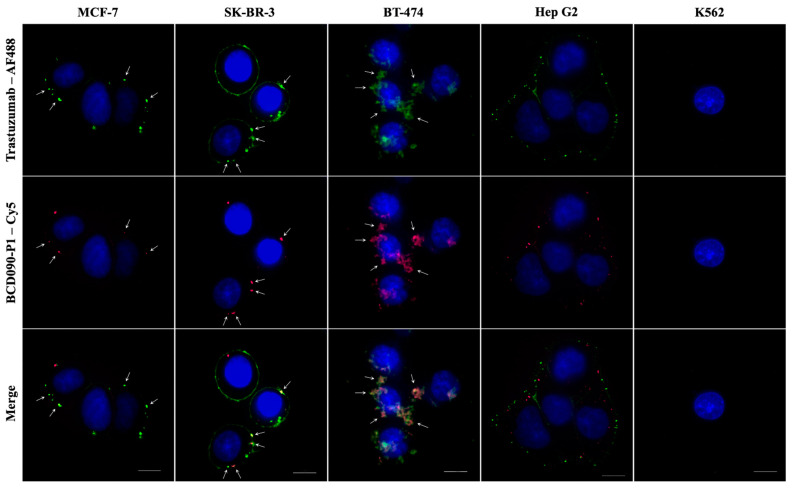
Immunofluorescent staining of cancer cells with anti-ErbB2 and anti-ErbB3 antibodies. Images obtained with trastuzumab or BCD090-P1 show similar staining patterns with most receptors localized in individual clusters presumably located on the plasma membrane and partly within intracellular vesicles. Non-epithelial K562 cells devoid of ErbB receptors were used as a negative control and did not show considerable staining with either antibody. The ErbB2 and ErbB3 receptors show remarkable co-localization in the breast adenocarcinoma cell lines MCF-7, SK-BR-3 and BT-474, but not in hepatocellular carcinoma Hep G2. White arrows indicate the ErbB2 and ErbB3 co-localization spots. Scale bars 10 μm.

To visualize the receptor binding previously established in surface plasmon resonance experiments, we performed immunocytochemical staining of several high ErbB3-expressing cancer cell lines with subsequent imaging by confocal microscopy. Non-epithelial K562 cells devoid of ErbB receptors were used as a negative control. We directly conjugated our most affine binder, BCD090-P1, and trastuzumab with Cy5 or AF488 fluorescent dyes with mean labeling efficiencies of two and seven dye molecules per antibody, respectively. The obtained images are presented in Figure 7. Staining with both antibodies produced a characteristic pattern with individual bright spots in all the ErbB3-overexpressing cells which we attribute to the localized ErbB3 and ErbB2 receptors, while no considerable staining with either antibody was observed in the K562 cells. The overall ErbB3 distribution pattern is similar to the one previously observed with anti-ErbB3 fluorophore-conjugated Affibodies [52], yet in our experiments the intracellular receptors are not detected because the microscopy was performed on live cells. In some cells, especially in SK-BR-3, the distribution of ErbB3 was so uneven that all the receptors accumulated in a single intense patch. We anticipate that stained ErbB2/ErbB3 receptors in MCF-7 cells are predominantly located on the plasma membrane, which is evident from the superposition of fluorescent and brightfield images (Figure S7). However, some fraction of ErbB3 is apparently located in intracellular vesicles, especially in Hep G2 cells, where red ErbB3–BCD090-P1 spots are closer to the nucleus than green ErbB2–trastuzumab complexes. It is unclear whether the internalization of ErbB3 in Hep G2 was stimulated by antibody binding, or the observed distribution reflects the natural receptor trafficking. The ErbB2 and ErbB3 receptors showed remarkable co-localization in the three breast adenocarcinoma cell lines MCF-7, SK-BR-3, and BT-474. The intense spots corresponding to receptor clusters frequently displayed perfect alignment in these cells, yet in BT-474 their appearance was more diffuse. In contrast, the ErbB2 and ErbB3 receptors in Hep G2 cells, although arranged in the same pattern of individual spots, showed no pronounced co-localization. The difference in receptor co-localization presumably reflects the distinct functional roles they have in hepatocellular carcinoma, as opposed to breast adenocarcinomas. The presented data demonstrate efficient ErbB3 targeting by the single-domain antibody in breast cancer cell cultures, and visualize the existence of isolated clusters of “oncogenic units” [6], where signaling through ErbB2/ErbB3 heterodimers does occur.

## 4. Discussion

The important role of ErbB3 in cancer progression stimulated the development of several monoclonal antibodies to block its aberrant signaling. All of these antibodies are classical immunoglobulins, and the only alternative scaffold used to inhibit ErbB3 is an Affibody, which is a small helix bundle protein derived from protein A [52,53]. In the present work, we took a different approach to targeting the ErbB3 receptor in cancer cells and studied new llama heavy-chain antibodies against its extracellular domain. The use of this special class of immunoglobulins expands the repertoire of anti-ErbB3 inhibitory antibodies, which may reveal novel therapeutically valuable epitopes and mechanisms of action.

The single-domain variable fragments were expressed in the cytoplasm of *E. coli* SHuffle cells as SUMO fusions, cleaved by TEV protease and purified to homogeneity, which allowed high yields of soluble protein without any tags remaining to be obtained. The characterization of single-domain antibodies included the analysis of their aggregation state, secondary structure, conformational stability, disulfide bonds, receptor binding, and antiproliferative action on cancer cells. Two antibodies, BCD090-P1 and BCD090-M2, noncompetitively interact with distinct epitopes on the receptor with nanomolar affinity and inhibit ErbB3-driven proliferation in cancer cells. They exert the antiproliferative action via two distinct mechanisms. While BCD090-M2 blocks ligand binding directly, BCD090-P1 does not compete with the ligand and presumably acts through a more complex allosteric mechanism.

We anticipate that llama antibodies possess several decisive advantages that can be realized both as full-length antibodies with an Fc fragment that is capable of stimulating an immune response, or as individual single-domain fragments with the utmost stability and ease of biotechnological production. First, they are particularly suitable for the design of bispecific antibodies against ErbB2/ErbB3 or a biparatopic antibody that would utilize the noncompetitive binding of BCD090-P1 and BCD090-M2 to distinct epitopes on ErbB3, to increase the avidity and inhibitory effect. Second, the single-domain antibodies present a perfect template for the production of antibody–drug conjugates, those efficiency against an ErbB3-expressing tumor was demonstrated with a conventional antibody linked to a plant toxin saporin [54]. Third, the small size and robust folding of single-domain antibodies allow their use in the form of intrabodies encoded by a therapeutic RNA [55], which can inhibit ErbB3 by interfering with its processing and trafficking before its appearance on the cell surface.

## Figures and Tables

**Table 1:** Equilibrium dissociation constants and kinetic rate constants obtained by the analysis of surface plasmon resonance data.

Antibody	k_a_ 10^−5^, M^−1^s^−1^	k_d_ ⋅10^3^, s^−1^	K_D_, nM from Kinetics	K_D_, nM from Equilibrium
BCD090-P1	19 ± 4	3.0 ± 1.0	1.6 ± 0.2	1.61 ± 0.06
BCD090-M2	2.2 ± 0.4	3.6 ± 0.1	16.5 ± 2.0	15 ± 1
BCD090-M456	0.0072 ± 0.0002	2.2 ± 0.2	3100 ± 200	8600 ± 800
pelB-BCD090-P1-His_5_	8.7 ± 2.0	4.4 ± 0.2	5.1 ± 0.9	6.5 ± 0.9

## Data Availability

The data obtained and presented in this article are available from the corresponding author upon reasonable request.

## Supplementary Materials

The following are available online at https://www.mdpi.com/article/10.3390/biomedicines9091106/s1: expression and purification of TEVpM2, periplasmic expression of BCD090-P1. Figure S1: plasmid maps; Figure S2: the purification of BCD090-P1 as monitored by SDS-PAGE; Figure S3: MS analysis of disulfide bonds in lysozyme and single-domain antibodies; Figure S4: surface plasmon resonance analysis of pelB-BCD090-P1-His5 binding to ErbB3 ECD; Figure S5: surface plasmon resonance analysis of antibody specificity; Figure S6: surface plasmon resonance analysis of BCD090-P1 binding to ErbB3 ECDIII; Figure S7: immunofluorescent staining of MCF-7 cells with trastuzumab and BCD090-P1. Table S1: expression vectors; Table S2: sequences of the fusion proteins and ErbB3 receptor fragments; Table S3: calculated physicochemical parameters of the single-domain antibodies and ErbB3 receptor fragments; Table S4: yield of the single-domain antibodies expressed in *E. coli* SHuffle and BL21(DE3); Table S5: thermodynamic parameters obtained from fitting of the urea denaturation curves; Table S6: free thiols and disulfides in single-domain antibodies as quantified by Ellman reagent and NTSB.
