# Supplementary material for: Targeting ErbB3 Receptor in Cancer with Inhibitory Antibodies from Llama
Source: Biomedicines. 2021 Aug 28;9(9):1106. doi: 10.3390/biomedicines9091106 (PMC8467012; doi:10.3390/biomedicines9091106)
Supplement: Supplementary file 1 [file biomedicines-09-01106-s001.zip › biomedicines-1317259-supplementary.pdf]

Expression and purification of TEVpM2

For the cleavage of fusion proteins, we used TEV protease mutant TEVpM2 developed by Wei et al. [1]. It is known that wild-type TEV protease undergoes auto-inactivation, which is inhibited by S219V mutation. TEVpM2 has S219V and five additional mutations (T17S, L56V, N68D, I77V, S135G), which greatly improve its solubility and expression in *E. coli*. To express TEVpM2, we used a pSol-His$_6$ vector from Expresso system (Lucigen). The TEVpM2 with N-terminal histidine tag was PCR-amplified from the original pET28 vector [1] using specific primers with Expresso overlap. The fragment was mixed with the linearized pSol-His$_6$ vector and used to transform chemically competent *E. cloni* 10G cells according to manufacturer recommendations. The construct was purified and used to transform *E. coli* BL21gold cells (Stratagene). The cells were cultured in 2xYT, at OD~0.8 protein expression was induced by the addition of *L*-rhamnose. After cells were lysed by ultrasonication, TEVpM2 was purified by a single IMAC step using HisTrap column, which resulted in > 80% purity. The buffer was exchanged to 20 mM Tris pH 8.0, 0.5 mM EDTA, 0.5 mM DTT, 50% glycerol. The 3 mg/ml solution of TEVpM2 was stored at -20 °C until use.

Periplasmic expression of BCD090-P1

E. coli BL21(DE3) cells (NEB) were transformed with the pET22-BCD090-P1 plasmid, and a single colony after selection with ampicillin was used to inoculate an overnight culture. The following day 10 mL of overnight culture were used to inoculate 1 liter of TB media containing 100 μg/mL ampicillin. Cells were grown at 37 °C until OD_{600} = 0.8–1.0. Protein expression was induced by the addition of IPTG to a final concentration of 0.1 mM, and cells were cultured for additional 5h at 30 °C. Then the periplasmic fraction was extracted via the classical cold osmotic shock method [2]. Cells were harvested by 10 min centrifugation at 4 000g and resuspended in 80 mL of room temperature solution A: 50 mM Tris pH 8.0, 20% sucrose, 0.1 mM EDTA. After 10 min incubation at room temperature, cells were centrifuged for 15 min at 12,500 g, the supernatant was collected, and the pellet was resuspended in 80 ml of ice-cold solution B: 0.5 mM $MgSO_4$. Cell suspension was incubated for an additional 10 min on ice with mild agitation and pelleted by 15 min centrifugation at 12,500 g. Both supernatant fractions A and B were combined and used for further purification, which proceeded essentially as described for cytoplasmic expression.

References

1. Wei, L.; Cai, X.; Qi, Z.; Rong, L.; Cheng, B.; Fan, J. In vivo and in vitro characterization of TEV protease mutants. *Protein Expr. Purif.* **2012**, *83*, 157–163, doi:10.1016/j.pep.2012.03.011.
2. Nossal, N.G.; Heppel, L.A. The Release of Enzymes by Osmotic Shock from Escherichia coli in Exponential Phase. *J. Biol. Chem.* **1966**, *241*, 3055–3062, doi:10.1016/s0021-9258(18)96497-5.

Table S1. Expression vectors.

Vector Name	Protein	Leader, Fusion Protein, Affinity Tags
pSolSUMO-BCD090-P1	BCD090-P1	N-term His_6, SUMO
pSolSUMO-BCD090-M2	BCD090-M2	N-term His_6, SUMO
pSolSUMO-BCD090-M456	BCD090-M456	N-term His_6, SUMO
pET22-BCD090-P1	BCD090-P1	N-term pelB leader, C-term His_5
pEE-ErbB3-ECD-His_6-FLAG	ErbB3-ECD	C-term His_6, C-term FLAG tag
pEE-ErbB3-ECD^{III}-His_6-EPEA	ErbB3-ECD^{III}	C-term His_6, C-term EPEA tag

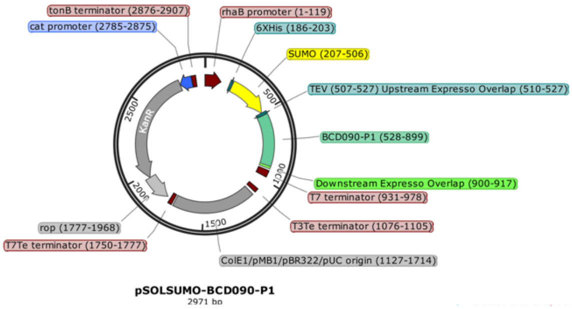
tonB terminator (2876-2907)
cat promoter (2785-2875)
rhaB promoter (1-119)
6XHis (186-203)
SUMO (207-506)
TEV (507-527) Upstream Expresso Overlap (510-527)
BCD090-P1 (528-899)
Downstream Expresso Overlap (900-917)
T7 terminator (931-978)
T3Te terminator (1076-1105)
ColE1/pMB1/pBR322/pUC origin (1127-1714)
rop (1777-1968)
T7Te terminator (1750-1777)
KanR
500
1000
1500
2000
2500
pSOLSUMO-BCD090-P1
2971 bp

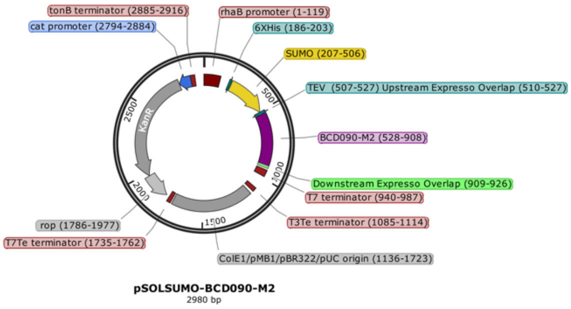
tonB terminator (2885-2916)
cat promoter (2794-2884)
rhaB promoter (1-119)
6XHis (186-203)
SUMO (207-506)
TEV (507-527) Upstream Expresso Overlap (510-527)
BCD090-M2 (528-908)
Downstream Expresso Overlap (909-926)
T7 terminator (940-987)
T3Te terminator (1085-1114)
ColE1/pMB1/pBR322/pUC origin (1136-1723)
rop (1786-1977)
T7Te terminator (1735-1762)
KanR
500
1000
1500
2000
2500
pSOLSUMO-BCD090-M2
2980 bp

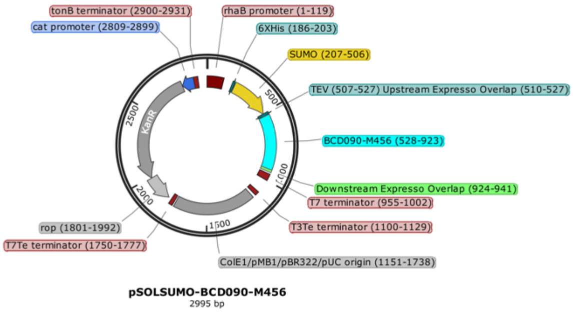
tonB terminator (2900-2931)
cat promoter (2809-2899)
rhaB promoter (1-119)
6XHis (186-203)
SUMO (207-506)
TEV (507-527) Upstream Expresso Overlap (510-527)
BCD090-M456 (528-923)
Downstream Expresso Overlap (924-941)
T7 terminator (955-1002)
T3Te terminator (1100-1129)
ColE1/pMB1/pBR322/pUC origin (1151-1738)
rop (1801-1992)
T7Te terminator (1750-1777)
KanR
500
1000
1500
2000
2500
pSOLSUMO-BCD090-M456
2995 bp

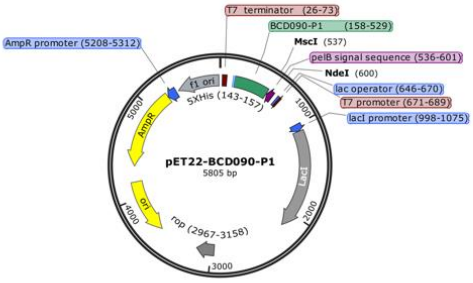
T7 terminator (26-73)
BCD090-P1 (158-529)
MscI (537)
pelB signal sequence (536-601)
NdeI (600)
lac operator (646-670)
T7 promoter (671-689)
lacI promoter (998-1075)
AmpR promoter (5208-5312)
f1 ori
5XHis (143-157)
AmpR
ori
rop (2967-3158)
LacI
pET22-BCD090-P1
5805 bp
1000
2000
3000
4000
5000

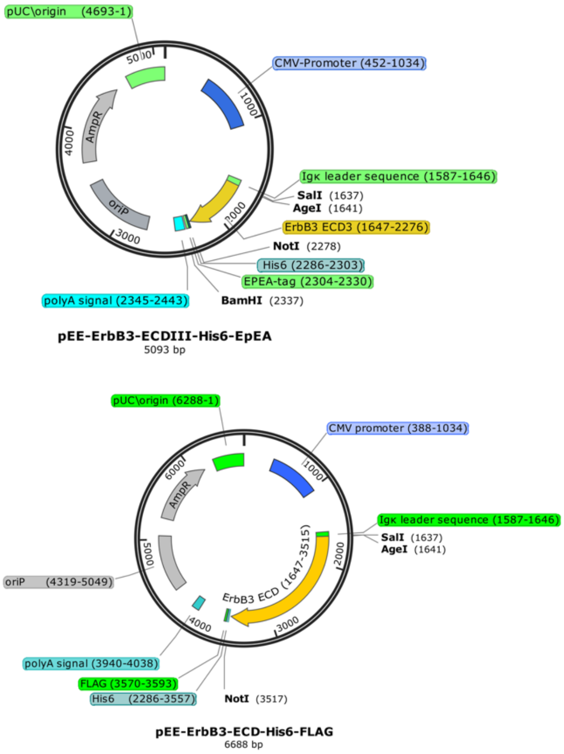

Figure S1. Plasmid maps.

Table S2. Sequences of the fusion proteins and ErbB3 receptor fragments.

Vector Name	Protein Sequence
pSolSUMO-BCD090-P1	**His-tag** 10 20 30 40 50 60<br>M**HHHHHH**MGS LQDSEVNQEA KPEVKPEVKP ETHINLKVSD GSSEIFFKIK KTTPLRRLME<br>70 80 90 100 110 **TEV** 120<br>AFAKRQGKEM DSLTFLYDGI EIQADQTPED LDMEDNDIIE AHREQIGG**EN** **LYFQG**EVQLV<br>130 140 150 160 170 180<br>QSGGGLVQPG GSLRLSCAAS GRTSSKYAMG WFRQAPGKGT EFVATISWSD GSTYYADSVE<br>190 200 210 220 230<br>GRFTISRDNA KNTVYLQMNS LKPEDTAVYY CAAAVDVLAG TFEYEYDYWG QGTLVTVSS<br>BCD090-P1
pSolSUMO-BCD090-M2	**His-tag** 10 20 30 40 50 60<br>M**HHHHHH**MGS LQDSEVNQEA KPEVKPEVKP ETHINLKVSD GSSEIFFKIK KTTPLRRLME<br>70 80 90 100 110 **TEV** 120<br>AFAKRQGKEM DSLTFLYDGI EIQADQTPED LDMEDNDIIE AHREQIGG**EN** **LYFQG**QVQLV<br>130 140 150 160 170 180<br>QSGGGLVQAG GSLRLSCAFS GRTFSMYTMG WFRQAPGKER EFVAANRGRG LSPDIADSVN<br>190 200 210 220 230 240<br>GRFTISRDNA KNTLYLQMDS LKPEDTAVYY CAADLQYGSS WPQRSSAEYD YWGQGTTVTV<br>SS BCD090-M2
pSolSUMO-BCD090-M456	**His-tag** 10 20 30 40 50 60<br>M**HHHHHH**MGS LQDSEVNQEA KPEVKPEVKP ETHINLKVSD GSSEIFFKIK KTTPLRRLME<br>70 80 90 100 110 **TEV** 120<br>AFAKRQGKEM DSLTFLYDGI EIQADQTPED LDMEDNDIIE AHREQIGG**EN** **LYFQG**QVQLV<br>130 140 150 160 170 180<br>QSGGGLVQSG GSLKLSCVAS GGPFSTYLMG WFRQAPGKER EFVTAISRSG LNTYYADSVK<br>190 200 210 220 230 240<br>GRFTISRDNA KNTVYLQMHS LKPEDTAVYH CAARRGGTNS GSYYFDRPAV SDEYDLWGQG<br>TLVTVSS BCD090-M456

pET22-BCD090-P1	10 **pelB** 20 30 40 50 60<br>**MKYLLPTAAA GLLLLAAQPA MA**MAEVQLVQ SGGGLVQPGG SLRLSCAASG RTSSKYAMGW<br>70 80 90 100 110 120<br>FRQAPGKGTE FVATISWSDG STYYADSVEG RFTISRDNAK NTVYLQMNSL KPEDTAVYYC<br>130 140 150 **His-tag**<br>AAAVDVLAGT FEYEYDYWGQ GTLVTVSS**HH HHH**<br><br>BCD090-P1
pEE-ErbB3-ECD-His_6-FLAG	10 20 30 40 50 60<br>EVGNSQAVCP GTLNGLSVTG DAENQYQTLY KLYERCEVVM GNLEIVLTGH NADLSFLQWI<br>70 80 90 100 110 120<br>REVTGYVLVA MNEFSTLPLP NLRVVRGTQV YDGKFAIFVM LNYNTNSSHA LRQLRLTQLT<br>130 140 150 160 170 180<br>EILSGGVYIE KNDKLCHMDT IDWRDIVRDR DAEIVVKDNG RSCPPCHEVC KGRCWGPGSE<br>190 200 210 220 230 240<br>DCQTLTKTIC APQCNGHCFG PNPNQCCHDE CAGGCSGPQD TDCFACRHFN DSGACVPRCP<br>250 260 270 280 290 300<br>QPLVYNKLTF QLEPNPHTKY QYGGVCVASC PHNFVVDQTS CVRACPPDKM EVDKNGLKMC<br>310 320 330 340 350 360<br>EPCGGLCPKA CEGTGSGSRF QTVDSSNIDG FVNCTKILGN LDFLITGLNG DPWHKIPALD<br>370 380 390 400 410 420<br>PEKLNVFRTV REITGYLNIQ SWPPHMHNFS VFSNLTTIGG RSLYNRGFSL LIMKNLNVTS<br>430 440 450 460 470 480<br>LGFRSLKEIS AGRIYISANR QLCYHHSLNW TKVLRGPTEE RLDIKHNSPR RDCVAEGKVC<br>490 500 510 520 530 540<br>DPLCSSGGCW GPGPGQCLSC RNYSRGGVCV THCNFLNGEP REFAHEAECF SCHPECQPME<br>550 560 570 580 590 600<br>GTATCNGSGS DTCAQCAHFR DGPHCVSSCP HGVLGAKGPI YKYPDVQNEC RPCHENCTQG<br>610 620 630 640 **FLAG-tag**<br>CKGPELQDCL GQTLVLIGKT HLTAAAGGGE S**HHHHHH**GDI L**DYKDDDDK**<br>**His-tag**<br>ErbB3 ECD (21-643)
pEE-ErbB3-ECD^{III}-His_6-EPEA	10 20 30 40 50 60<br>SGDKACEGTG SGSRFQTVDS SNIDGFVNCT KILGNLDFLI TGLNGDPWHK IPALDPEKLN<br>70 80 90 100 110 120<br>VFRTVREITG YLNIQSWPPH MHNFSVFSNL TTIGGRSLYN RGFSLLIMKN LNVTSLGFRS<br>130 140 150 160 170 180<br>LKEISAGRIY ISANRQLCYH HSLNWTKVLR GPTEERLDIK HNRPRRDCVA EGKVCDPLCS<br>190 200 210 220 **EPEA-tag**<br>SGGCWGPGPG QCLSCRNYSR GGVCVTHGNS AAA**HHHHHH**G YQDY**EPEA**<br>**His-tag**<br>ErbB3 ECD^{III} (329-532)

Kabat numbering and CDRs

Kabat number	1	2	3	4	5	6	7	8	9	10	11	12	13	14	15	16	17	18	19	20
BCD090-P1	E	V	Q	L	V	Q	S	G	G	G	L	V	Q	P	G	G	S	L	R	L
BCD090-M2	Q	V	Q	L	V	Q	S	G	G	G	L	V	Q	A	G	G	S	L	R	L
BCD090-M456	Q	V	Q	L	V	Q	S	G	G	G	L	V	Q	S	G	G	S	L	K	L

CDR H1 (Kabat)

Kabat number	21	22	23	24	25	26	27	28	29	30	**31**	**32**	**33**	**34**	**35**	36	37	38	39	40
BCD090-P1	S	C	A	A	S	G	R	T	S	S	**K**	**Y**	**A**	**M**	**G**	W	F	R	Q	A
BCD090-M2	S	C	A	F	S	G	R	T	F	S	**M**	**Y**	**T**	**M**	**G**	W	F	R	Q	A
BCD090-M456	S	C	V	A	S	G	G	P	F	S	**T**	**Y**	**L**	**M**	**G**	W	F	R	Q	A

CDR H2 (Kabat)

Kabat number	41	42	43	44	45	46	47	48	49	**50**	**51**	**52**	**52^{A}**	**53**	**54**	**55**	**56**	**57**	**58**	**59**	**60**
BCD090-P1	P	G	K	G	T	E	F	V	A	**T**	**I**	**S**	**W**	**S**	**D**	**G**	**S**	**T**	**Y**	**Y**	**A**
BCD090-M2	P	G	K	E	R	E	F	V	A	**A**	**N**	**R**	**G**	**R**	**G**	**L**	**S**	**P**	**D**	**I**	**A**
BCD090-M456	P	G	K	E	R	E	F	V	T	**A**	**I**	**S**	**R**	**S**	**G**	**L**	**N**	**T**	**Y**	**Y**	**A**

Kabat number	61	62	63	64	65	66	67	68	69	70	71	72	73	74	75	76	77	78	79	80
BCD090-P1	**D**	**S**	**V**	**E**	**G**	R	F	T	I	S	R	D	N	A	K	N	T	V	Y	L
BCD090-M2	**D**	**S**	**V**	**N**	**G**	R	F	T	I	S	R	D	N	A	K	N	T	L	Y	L
BCD090-M456	**D**	**S**	**V**	**K**	**G**	R	F	T	I	S	R	D	N	A	K	N	T	V	Y	L

Kabat number	81	82	82^A	82^B	82^C	83	84	85	86	87	88	89	90	91	92	93	94	95	96	97	98	99	100
BCD090-P1	Q	M	N	S	L	K	P	E	D	T	A	V	Y	Y	C	A	A	**A**	**V**	**D**	**V**	**L**	**A**
BCD090-M2	Q	M	D	S	L	K	P	E	D	T	A	V	Y	Y	C	A	A	**D**	**L**	**Q**	**Y**	**G**	**S**
BCD090-M456	Q	M	H	S	L	K	P	E	D	T	A	V	Y	H	C	A	A	**R**	**R**	**G**	**G**	**T**	**N**

CDR H3 (Kabat)

Kabat number	100^A	100^B	100^C	100^D	100^E	100^F	100^G	100^H	100^I	100^J	100^K	100^L	100^M	100^N	100°
BCD090-P1	**G**	**T**	**F**	**E**	**Y**	**E**	**Y**	-	-	-	-	-	-	-	-
BCD090-M2	**S**	**W**	**P**	**Q**	**R**	**S**	**S**	**A**	**E**	**Y**	-	-	-	-	-
BCD090-M456	**S**	**G**	**S**	**Y**	**Y**	**F**	**D**	**R**	**P**	**A**	**V**	**S**	**D**	**E**	**Y**

Kabat number	101	102	103	104	105	106	107	108	109	110	111	112	113
BCD090-P1	**D**	**Y**	W	G	Q	G	T	L	V	T	V	S	S
BCD090-M2	**D**	**Y**	W	G	Q	G	T	T	V	T	V	S	S
BCD090-M456	**D**	**L**	W	G	Q	G	T	L	V	T	V	S	S

Table S3. Calculated physicochemical parameters of the single-domain antibodies and ErbB3 receptor fragments.

Protein	Length, a.a.	ε_{280}, $M^{-1}cm^{-1}$	Formula		Molecular Weight (av.)		pI
			all S-H	all S-S	all S-H	all S-S	
BCD090-P1	125	$30 \cdot 10^3$	$C_{594}H_{898}N_{156}O_{192}S_4$	$C_{594}H_{896}N_{156}O_{192}S_4$	13424.7	13422.7	4.76
BCD090-M2	128	$27 \cdot 10^3$	$C_{609}H_{932}N_{174}O_{194}S_5$	$C_{609}H_{930}N_{174}O_{194}S_5$	13955.3	13953.3	7.96
BCD090-M456	133	$23 \cdot 10^3$	$C_{632}H_{973}N_{179}O_{198}S_4$	$C_{632}H_{971}N_{179}O_{198}S_4$	14374.9	14372.8	8.96
pelB-BCD090-P1-His_5	153	$31.5 \cdot 10^3$	$C_{733}H_{1116}N_{196}O_{223}S_7$	$C_{733}H_{1114}N_{196}O_{223}S_7$	16466.4	16464.4	6.17
ECD-His_6-FLAG	649	$68.4 \cdot 10^3$	$C_{3072}H_{4769}N_{899}O_{946}S_{59}$	$C_{3072}H_{4719}N_{899}O_{946}S_{59}$	71323.1	71272.7	6.24
ECD^{III}-His_6-EPEA	228	$33 \cdot 10^3$	$C_{1102}H_{1705}N_{333}O_{327}S_{12}$	$C_{1102}H_{1695}N_{333}O_{327}S_{12}$	25235.2	25225.1	8.69

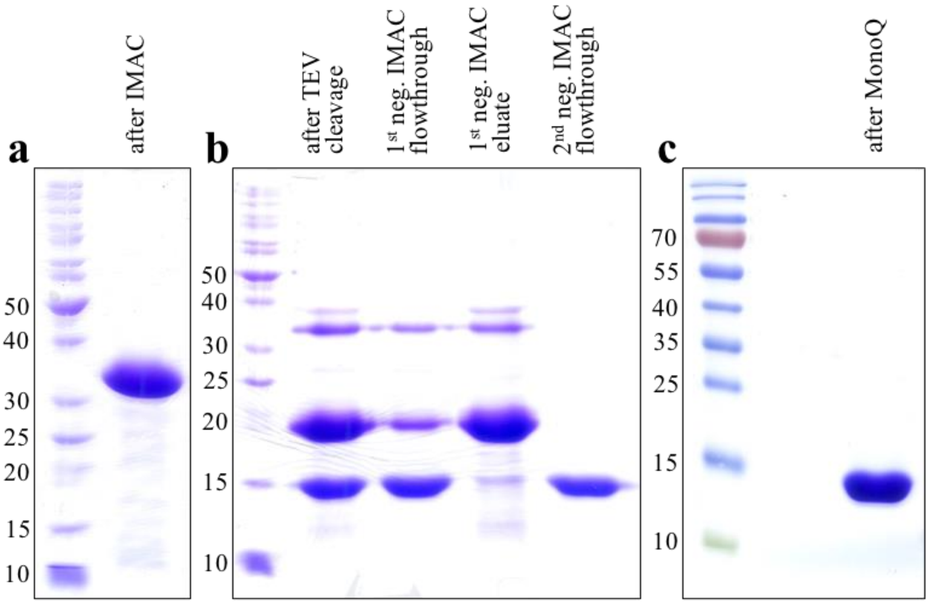

Figure S2. The purification of BCD090-P1 as monitored by SDS-PAGE. a) the fusion protein after purification by IMAC, the recombinant fusion protein have molecular mass ~27 kDa but migrates

anomalously near 35 kDa due to histidine tag and SUMO; b) analysis of the TEV cleavage products and negative IMAC purification, untagged antibody migrates near 15 kDa band as expected, His_6-SUMO migrates anomalously near 20 kDa standard; c) BCD090-P1 analyzed after polishing step of high-resolution anion exchange chromatography.

Table S4. Yield of the single-domain antibodies expressed in *E. coli* SHuffle and BL21(DE3). Protein quantitation on each purification step was done spectrophotometrically using calculated extinction coefficient at 280 nm. The values are reported per 1 liter of cell culture.

	***E. coli* SHuffle / *E. coli* BL21(DE3)**		
	**BCD090-P1**	**BCD090-M2**	**BCD090-M456**
Final OD_{600}	9.0/12.4	8.8/9.7	8.7/9.5
Wet cell weight, g	14.0/20.8	14.4/18.0	12.8/18.4
Fusion protein after IMAC, mg	146/266	48/–*	184/118
Untagged antibody after TEV and neg. IMAC, mg	58	16	72
Untagged antibody after MonoQ/S, mg	49	13	60

*No soluble protein was obtained

Table S5. Thermodynamic parameters obtained from fitting of the urea denaturation curves.

**Protein**	**$[Urea]_{1/2}$, M**	**Slope (m), Kcal mol^{-1} M^{-1}**	**ΔG_{H2O}, Kcal mol^{-1}**
BCD090-P1	7.60 ±0.05	1.54 ±0.19	11.7 ±1.4
BCD090-M2	4.37 ±0.01	1.65 ±0.03	7.2 ±0.16
BCD090-M456	5.85 ±0.07	1.17 ±0.13	6.9 ±0.8
pelB-BCD090-P1-His_5	8.20 ±0.05	1.10 ±0.10	9.1 ±0.8

Table S6. Free thiols and disulfides in single-domain antibodies as quantified by Ellman reagent and NTSB. Hen egg lysozyme was used as a reference in both experiments. Zeroes indicate samples where no measurable changes in absorption were detected in all replicates.

**Protein**	**S-H, Theoretical**	**S-H, Ellman**	**S-S, Theoretical**	**S-S (½ total Cys), Na_2SO_3 + NTSB**
BCD090-P1	0	0	1	0.96 ±0.03
BCD090-M2	0	0	1	1.00 ±0.01
BCD090-M456	0	0.97 ±0.07	1	0.83 ±0.1
Lysozyme	0	0	4	3.1 ±0.4

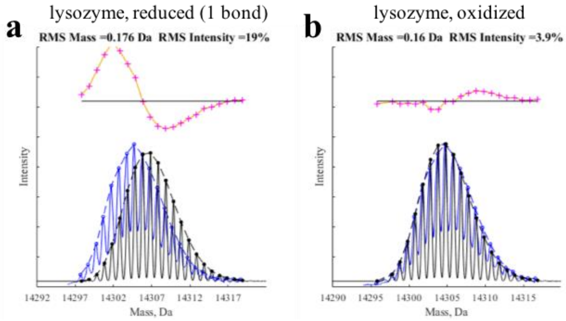

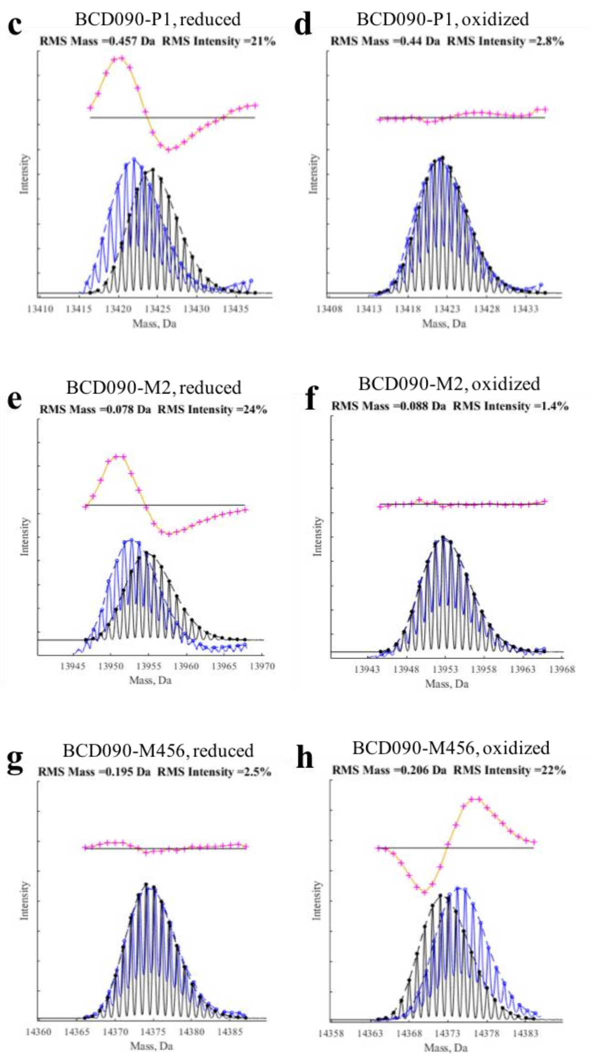

Figure S3. MS analysis of disulfide bonds in lysozyme and single-domain antibodies. Experimental ESI-MS data after charge deconvolution shown in blue and theoretical isotopic distributions are black. For each protein, experimental data were fitted with a formula corresponding either to a single reduced disulfide bond (panels a, c, e, g) or oxidized cysteines (panels b, d, f, h). As seen from the graphs, for all proteins except BCD090-M456 experimental data evidence the existence of disulfide bonds. In contrast, MS spectra for BCD090-M456 are consistent with both cysteines in a reduced state.

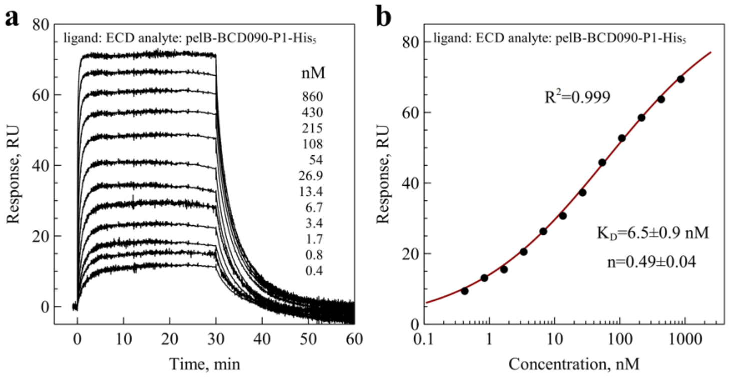

Figure S4. Surface plasmon resonance analysis of pelB-BCD090-P1-His5 binding to ErbB3 ECD. (**a**) experimental binding sensograms; (**b**) equilibrium binding response (dots) fitted with Hill equation (line). The analysis gives K_D = 6.5 nM.

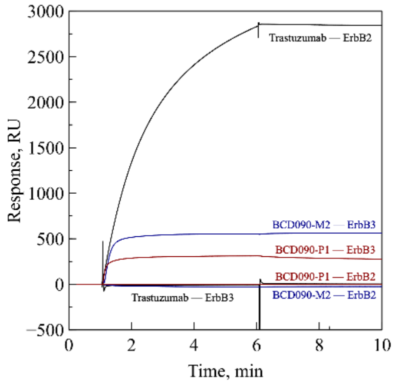

Figure S5. Surface plasmon resonance analysis of antibody specificity. Recombinant human ErbB2 ECD fused to Fc fragment was produced in CHO cells essentially as described for ErbB3. Two receptors were immobilized at the adjacent lanes of CM5 sensor chip. BCD090-P1 and BCD090-M2 were injected at 0.5 μM, and trastuzumab was injected at 0.1 μM concentration. In contrast to trastuzumab, both single-domain antibodies showed no measurable binding to ErbB2.

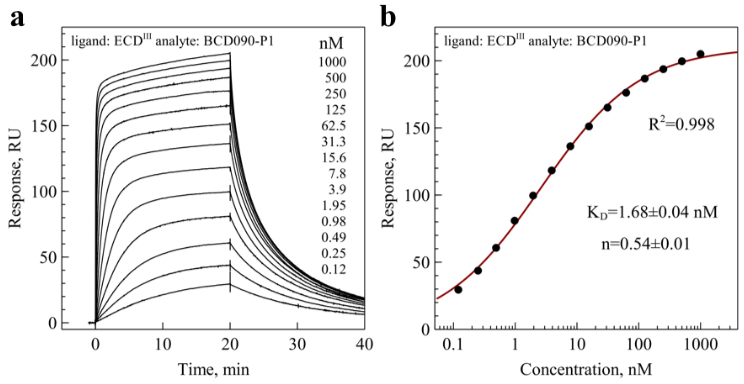

Figure S6. Surface plasmon resonance analysis of BCD090-P1 binding to ErbB3 ECD^{III}. (**a**) experimental binding sensograms; (**b**) equilibrium binding response (dots) fitted with Hill equation (line). The analysis gives K_D = 1.68 nM.

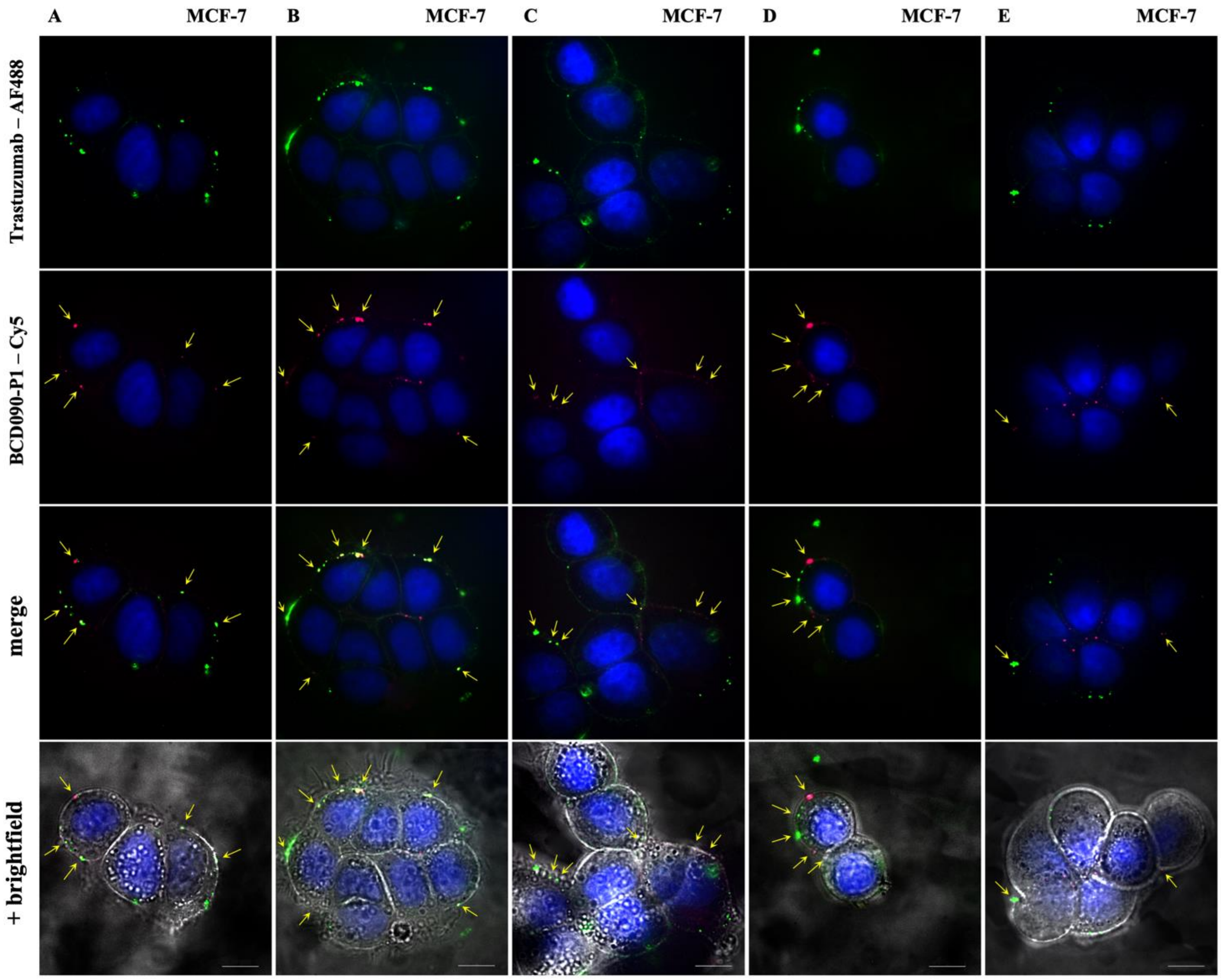

Figure S7. Immunofluorescent staining of MCF-7 cells with trastuzumab and BCD090-P1. Yellow arrows indicate ErbB3 spots located presumably on the plasma membrane. Brightfield images included to visualize membrane localization of the receptor.
